# Association of Prenatal Ultrasonographic Findings With Adverse Neonatal Outcomes Among Pregnant Women With Zika Virus Infection in Brazil

**DOI:** 10.1001/jamanetworkopen.2018.6529

**Published:** 2018-12-28

**Authors:** Jose Paulo Pereira, Karin Nielsen-Saines, Jeffrey Sperling, Melanie M. Maykin, Luana Damasceno, Renan Fonseca Cardozo, Helena Abreu Valle, Beatriz Ribeiro Torres Dutra, Helder Dotta Gama, Kristina Adachi, Andrea A. Zin, Irena Tsui, Zilton Vasconcelos, Patricia Brasil, Maria E. Moreira, Stephanie L. Gaw

**Affiliations:** 1 Instituto Nacional de Saúde da Mulher, da Criança e do Adolescente Fernandes Figueira–Fundação Oswaldo Cruz, Rio de Janeiro, Brazil; 2 Division of Pediatric Infectious Diseases, Department of Pediatrics, University of California, Los Angeles; 3 Division of Maternal-Fetal Medicine, Department of Obstetrics, Gynecology, and Reproductive Sciences, University of California, San Francisco; 4 Laboratorio de Doenças Febris Agudas, Instituto de Infectologia Evandro Chagas–Fundação Oswaldo Cruz, Rio de Janeiro, Brazil; 5 Jules Stein Eye Institute, Retina Division, UCLA (University of California, Los Angeles)

## Abstract

**Importance:**

Congenital Zika virus infection causes a spectrum of adverse birth outcomes, including severe birth defects of the central nervous system. The association of prenatal ultrasonographic findings with adverse neonatal outcomes, beyond structural anomalies such as microcephaly, has not been described to date.

**Objective:**

To determine whether prenatal ultrasonographic examination results are associated with abnormal neonatal outcomes in Zika virus–affected pregnancies.

**Design, Setting, and Participants:**

A prospective cohort study conducted at a single regional referral center in Rio de Janeiro, Brazil, from September 1, 2015, to May 31, 2016, among 92 pregnant women diagnosed during pregnancy with Zika virus infection by reverse-transcription polymerase chain reaction, who underwent subsequent prenatal ultrasonographic and neonatal evaluation.

**Exposures:**

Prenatal ultrasonography.

**Main Outcomes and Measures:**

The primary outcome measure was composite adverse neonatal outcome (perinatal death, abnormal finding on neonatal examination, or abnormal finding on postnatal neuroimaging). Secondary outcomes include association of specific findings with neonatal outcomes.

**Results:**

Of 92 mother-neonate dyads (mean [SD] maternal age, 29.4 [6.3] years), 55 (60%) had normal results and 37 (40%) had abnormal results on prenatal ultrasonographic examinations. The median gestational age at delivery was 38.6 weeks (interquartile range, 37.9-39.3). Of the 45 neonates with composite adverse outcome, 23 (51%) had normal results on prenatal ultrasonography. Eleven pregnant women (12%) had a Zika virus–associated finding that was associated with an abnormal result on neonatal examination (adjusted odds ratio [aOR], 11.6; 95% CI, 1.8-72.8), abnormal result on postnatal neuroimaging (aOR, 6.7; 95% CI, 1.1-38.9), and composite adverse neonatal outcome (aOR, 27.2; 95% CI, 2.5-296.6). Abnormal results on middle cerebral artery Doppler ultrasonography were associated with neonatal examination abnormalities (aOR, 12.8; 95% CI, 2.6-63.2), postnatal neuroimaging abnormalities (aOR, 8.8; 95% CI, 1.7-45.9), and composite adverse neonatal outcome (aOR, 20.5; 95% CI, 3.2-132.6). There were 2 perinatal deaths. Abnormal findings on prenatal ultrasonography had a sensitivity of 48.9% (95% CI, 33.7%-64.2%) and a specificity of 68.1% (95% CI, 52.9%-80.1%) for association with composite adverse neonatal outcomes. For a Zika virus–associated abnormal result on prenatal ultrasonography, the sensitivity was lower (22.2%; 95% CI, 11.2%-37.1%) but the specificity was higher (97.9%; 95% CI, 88.7%-99.9%).

**Conclusions and Relevance:**

Abnormal results on prenatal ultrasonography were associated with adverse outcomes in congenital Zika infection. The absence of abnormal findings on prenatal ultrasonography was not associated with a normal neonatal outcome. Comprehensive evaluation is recommended for all neonates with prenatal Zika virus exposure.

## Key Points

**Question:**

Are prenatal ultrasonographic findings in maternal Zika virus infection associated with adverse neonatal outcomes?

**Findings:**

In this cohort study of 92 women with confirmed Zika virus infection in pregnancy, 37 had an abnormal result on prenatal ultrasonography that was associated with adverse composite neonatal outcomes. However, 23 of 55 neonates who had normal results on prenatal ultrasonography still had adverse neonatal outcomes.

**Meaning:**

Abnormal results on prenatal ultrasonography are associated with adverse neonatal outcomes; however, a comprehensive neonatal evaluation is recommended for all infants with suspected in utero Zika exposure.

## Teaser

This cohort study examines whether prenatal ultrasonographic results are associated with abnormal neonatal outcomes in Zika virus–affected pregnancies in Brazil.

## Introduction

The rate of abnormal perinatal outcomes after maternal Zika virus infection has been estimated to be from 6% to 55% for infections acquired in the first trimester and from 3% to 29% for infections acquired in the third trimester.^1^ The spectrum of anomalies associated with maternal Zika virus infection is still being characterized, and most information to date has been gleaned from retrospective cohorts and case-control studies.^2,3,4,5,6,7,8,9,10^ Recent reports evaluating the predictive value of prenatal ultrasonography are limited because they included cohorts identified prenatally or postnatally with microcephaly alone and did not include all pregnant women infected with Zika virus.^11,12^ Similarly, variation in the case definition of Zika virus infection by different groups, particularly the reliance on serologic diagnosis in endemic areas, complicates the interpretation of results owing to the cross-reactivity of antibodies with other common flaviviruses, such as dengue virus.^13,14^ Although microcephaly was among the birth defects initially associated with congenital Zika virus, additional brain abnormalities have been identified in its absence, and the spectrum of postnatal abnormalities is not yet fully characterized.^10,15,16,17,18,19^ Reports have primarily focused on the predictive value of prenatal ultrasonography in identifying microcephaly among affected neonates.^20^ However, it is likely that not all Zika virus–infected fetuses will be affected to the same degree, such as with cytomegalovirus infection^21^; thus, focusing prenatal diagnosis on the detection of microcephaly will fail to identify all infants at risk for adverse outcome.

As such, the challenge for clinicians is to identify which maternal Zika virus infections will have an abnormal neonatal outcome.^1,22^ Prenatal screening of potentially infected fetuses is critical for patient counseling of pregnancy care options, as well as optimizing the delivery setting and care of the neonate. However, the association of prenatal ultrasonographic findings with neonatal outcomes from Zika virus infection remains to be fully defined. The aim of this study was to assess the association between prenatal ultrasonographic findings and neonatal outcomes among pregnant women with confirmed Zika virus infection.

## Methods

### Study Design and Participants

This was a nested, prospective cohort study evaluating the clinical manifestations and neonatal outcomes of symptomatic Zika virus infection during pregnancy. Data were reported according to the Strengthening the Reporting of Observational Studies in Epidemiology (STROBE) reporting guidelines for cohort studies. This study was approved by the institutional review boards at Fundação Oswaldo Cruz (Fiocruz), Rio de Janeiro, Brazil, and the University of California, Los Angeles, and considered exempt at the University of California, San Francisco. Written informed consent was obtained from each patient at the time of enrollment. From September 1, 2015, through May 31, 2016, 345 pregnant women who presented to Fundação Oswaldo Cruz with a rash the previous 5 days were eligible and included after providing informed consent. Details of the initial cohort have been previously described.^2^ No fetal malformations were identified before study enrollment, and the study population was without chronic medical conditions. All of the patients underwent serologic or molecular testing for dengue, chikungunya, measles, parvovirus B19, cytomegalovirus, HIV, and syphilis as part of the study protocol and through routine prenatal care through Brazilian guidelines. In this study, we included women with a positive result for Zika virus confirmed by serum or urine reverse-transcription polymerase chain reaction (RT-PCR) who underwent at least 1 prenatal ultrasonography session after infection and had known neonatal outcomes. All infants were born and examined at Instituto Fernandes Figueira, Rio de Janeiro, Brazil, a tertiary maternal and pediatric referral center. Gestational age was confirmed by the earliest ultrasonographic measurements available (either through prenatal records or at the study site) in all patients.

### Procedures

All patients who were included in the study were referred for prenatal ultrasonography at enrollment, between 20 and 30 weeks’ gestation, and after 30 weeks’ gestation per national guidelines. Perinatologists certified by the Brazilian College of Radiology and Diagnostic Imaging and the Brazilian Federation of Gynecology and Obstetrics Associations (FEBRASGO) performed all prenatal ultrasonography with a 4-mHz to 8-mHz probe (Voluson 730 Expert or Voluson E6; GE Healthcare). Standard fetal biometric measurements (biparietal diameter, head circumference, abdominal circumference, and femur length) were obtained as well as an anatomic survey of the fetus as previously described. The Hadlock formula and standards were used to define fetal growth restriction (fetal weight <10th percentile) and macrosomia (fetal weight >90th percentile) for gestational age.^23^ Microcephaly was defined as fetal head circumference less than 2 SDs below the mean for a given gestational age or below the third percentile for gestational age as per standards set by the Brazilian Ministry of Health.^24^ Ventriculomegaly was defined as the downside lateral ventricle at the level of the atria measuring 10 mm or more. Mega cisterna magna was defined as a cisterna magna measurement greater than 10 mm on the oblique transverse plane in the setting of normal cerebellar hemispheres and vermis. Oligohydramnios and polyhydramnios were defined as a deepest vertical pocket less than 2 cm and greater than 8 cm, respectively. Placentomegaly was defined as greater than 4 cm in maximal placenta thickness. For Doppler studies, the pulsatility indexes of the umbilical artery and middle cerebral artery (MCA) were defined as abnormal if greater than the 95th percentile or if the peak systolic velocity of the MCA was greater than 1.5 multiples of the median.

Detailed prenatal ultrasonographic data were collected prospectively and abstracted for each patient. Each patient was considered as a single case, irrespective of the number of ultrasonographic scans performed. The ultrasonographic result was defined as abnormal if any of the following was noted on at least 1 examination: any structural anomaly, abnormal fetal growth measurements (fetal growth restriction or macrosomia), abnormal umbilical artery or MCA Doppler measurements, abnormal amniotic fluid assessment (oligohydramnios or polyhydramnios), or placentomegaly. Abnormal results found on ultrasonography were then categorized as a Zika-associated abnormality or an ultrasonographic finding of unknown significance in Zika infection. Zika-associated ultrasonographic findings included any major central nervous system (CNS) abnormality (microcephaly, calcifications, ventriculomegaly, Blake pouch cyst, cerebellar vermis hypoplasia, or agenesis of the corpus callosum), fetal growth restriction, or arthrogryposis. Ultrasonographic findings of unknown significance in Zika infection included those not previously described in Zika infection and of unclear clinical significance if they were to be identified in isolation, such as abnormal Doppler results, amniotic fluid abnormalities, macrosomia, placentomegaly, mega cisterna magna, and others.

Perinatal data collected at the time of birth included gestational age at delivery, mode of delivery, neonatal intensive care unit (NICU) admission, duration of NICU admission, and perinatal death. All neonates had physical examinations performed by pediatricians from the study team, including neonatologists, pediatric infectious disease specialists, geneticists, and neurologists. Funduscopic eye examinations were performed by trained pediatric ophthalmologists as previously described.^25^ Hearing assessments were performed through brainstem evoked response audiometry. Anthropometric measures at birth were obtained for all live births as previously described, and microcephaly at birth was defined as a head circumference *z* score less than −2.^2^ Small and large for gestational age were defined per International Fetal and Newborn Growth Consortium for the 21st Century (INTERGROWTH-21st) standards.^26^ Neuroimaging was performed at the clinician’s discretion or patient preference by transfontanelle ultrasonography, computerized tomography of the brain, or brain magnetic resonance imaging. Not all infants underwent eye, hearing, or neuroimaging studies. The primary outcome was a composite adverse neonatal outcome, defined as perinatal death (stillbirth or death within 28 days of life), an abnormal finding on neonatal examination, or an abnormal finding on postnatal neuroimaging.

### Statistical Analysis

We analyzed the association between prenatal ultrasonographic findings and neonatal outcomes. Secondary outcomes were associations between abnormal results on ultrasonography and adverse neonatal outcomes at neonatal examination or abnormal postnatal neuroimaging results. Categorical variables were compared with the Fisher exact test or χ^2^ test, and continuous variables were compared using a 2-tailed *t* test (parametric) or Wilcoxon rank sum test (nonparametric). Adjusted odds ratios (aORs) and 95% CIs were calculated by multivariate logistic regression. All *P* values were 2-sided and were considered statistically significant if less than .05. Statistical analysis was performed using Stata, version 14.2 (StataCorp). When data were missing or not obtained, the appropriate denominator was indicated in the tables.

## Results

During the study period, 182 pregnant women (mean [SD] maternal age at enrollment, 29.4 [6.3] years) had positive results for Zika virus confirmed by RT-PCR and were offered ultrasonographic examination, of whom 92 pregnant women (51%) opted for at least 1 prenatal ultrasonographic examination and had known neonatal outcomes. The most common reason stated for declining prenatal ultrasonography was fear of discovering a fetal anomaly with no ability to change the pregnancy outcome. Of the 92 mother-neonate dyads included in the final analysis, 55 pregnant women (60%) had normal results on ultrasonography and 37 pregnant women (40%) had at least 1 abnormal result on ultrasonography (Figure). In patients with normal results on prenatal ultrasonography, all pregnancies resulted in live births, from which 23 of 55 neonates (42%) had an adverse neonatal outcome. Of the 37 patients with abnormal findings on prenatal ultrasonography, there was 1 fetal death at 36 weeks’ gestation, and 21 of 36 neonates (58%) had an abnormal outcome, including 1 neonatal death on day 1 of life.

**Figure.**
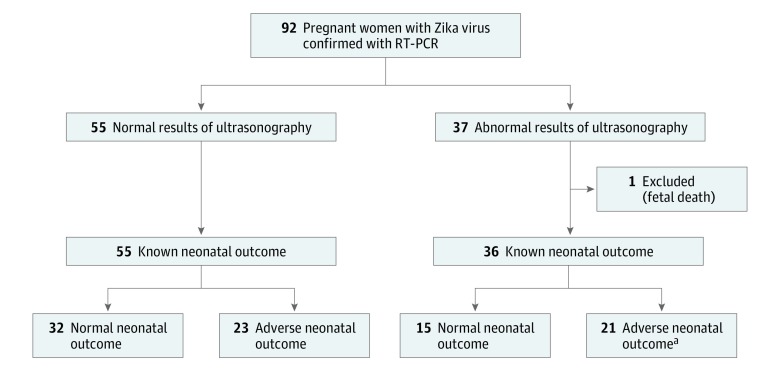
Flow of Patients Through the Study Cases with abnormal ultrasonographic findings have any major or minor ultrasonographic finding on at least 1 ultrasonographic examination during the pregnancy. Abnormal pregnancy outcome was defined as a case with any of the following: perinatal death, abnormal neonatal examination results, or abnormal postnatal neuroimaging results. RT-PCR indicates reverse transcription–polymerase chain reaction. ^a^Includes 1 neonatal death on day of life 1.

The spectrum and classification of prenatal ultrasonographic findings are shown in Table 1. Thirty-seven of the 92 pregnant women (40%) were found to have at least 1 abnormal finding on prenatal ultrasonography. Ultrasonographic findings were grouped into Zika-associated abnormalities and findings of unknown significance in Zika infection. The most common Zika-associated abnormalities were CNS abnormalities (10 of 92 fetuses [11%]), including microcephaly, calcifications, and ventriculomegaly. Abnormal umbilical artery and MCA Doppler abnormalities were also found in 5 (5%) and 16 (17%) fetuses, respectively, and were sometimes transiently abnormal. There were 26 fetuses with an isolated ultrasonographic finding of unknown significance. All cases with a Zika-associated abnormal result on ultrasonography had at least 1 ultrasonographic finding with no prior association with Zika infection, most commonly abnormal Doppler measurements.

**Table 1.** Classification of Abnormal Findings on Prenatal Ultrasonography Among 92 Women Diagnosed During Pregnancy With Zika Virus Infection

Classification of Abnormal Prenatal Ultrasonographic Findings	No. of Pregnant Women (% of Total Cohort)^a^
Any abnormal finding	37 (40)
Zika virus–associated abnormal finding	11 (12)
Major CNS abnormality	10 (11)
Microcephaly	7 (8)
Calcifications	9 (10)
Ventriculomegaly	6 (7)
Blake pouch cyst	3 (3)
Cerebellar vermis hypoplasia	3 (3)
Agenesis of the corpus callosum	2 (2)
Fetal growth restriction	7 (8)
Arthrogryposis	1 (1)
Findings of unknown significance	37 (40)
Minor result only	26 (28)
Abnormal Doppler examination finding	17 (18)
Umbilical artery	5 (5)
Middle cerebral artery	16 (17)
Fluid abnormalities	13 (14)
Oligohydramnios	6 (7)
Polyhydramnios	7 (8)
Placentomegaly	11 (12)
Macrosomia	8 (9)
Mega cisterna magna	4 (4)
Other^b^	8 (9)

Abbreviation: CNS, central nervous system.

^a^ The total cohort was 92 pregnant women. Pregnant women may have more than 1 finding.

^b^ Included pelviectasis (n = 2), brachycephaly (n = 2), hypoechogenic area in chest (n = 2), choroid plexus cyst (n = 1), and liver calcification (n = 1).

Comparison of baseline characteristics between pregnant women with normal results on ultrasonography vs those with abnormal results on ultrasonography revealed that pregnant women with abnormal results on ultrasonography were younger (mean [SD] age, 27.8 [6.5] years for pregnant women with normal ultrasonographic results vs 30.5 [6.1] years for pregnant women with abnormal ultrasonographic results; *P* = .05) and underwent more frequent ultrasonographic examinations (mean [SD] of 3.5 [1.9] examinations for pregnant women with normal ultrasonographic results vs 2.6 [1.3] examinations for pregnant women with abnormal ultrasonographic results; *P* = .01) (eTable 1 in the Supplement). There were no significant differences in gestational age at the time of Zika virus infection between the 2 groups. One pregnant woman had a positive test result for co-infection with chikungunya virus by RT-PCR; this pregnant woman had a normal result on prenatal ultrasonography and a normal neonatal outcome. There were no differences in serologic evidence of other congenital infections, including chikungunya virus, cytomegalovirus, dengue, measles, syphilis, and toxoplasmosis (data not shown).

Perinatal outcomes for the 91 liveborn infants in the 2 groups (normal ultrasonographic results group and abnormal ultrasonographic results group) are shown in Table 2. The median gestational age at delivery was 38.6 weeks (interquartile range, 37.9-39.3 weeks). There were no significant between-group differences in gestational age at delivery, neonate sex, preterm birth, birth weight, or emergency cesarean delivery. The abnormal ultrasonographic results group was more likely to require admission to the NICU (11 of 36 neonates [31%] in the abnormal ultrasonographic results group vs 7 of 54 [13%] in the normal ultrasonographic results group; *P* = .04). Most NICU admissions were for full-term infants born after 37 weeks’ gestation. There were 6 cases of neonatal microcephaly, all of which were detected on prenatal ultrasonography. Both cases of perinatal death had an abnormal finding on prenatal ultrasonography.

**Table 2.** Neonatal Outcomes According to Ultrasonographic Findings Among Women Diagnosed During Pregnancy With Zika Virus Infection^a^

Neonatal Outcome	Prenatal Ultrasonographic Finding, No./Total No. (%)		*P* Value^b^
	Normal (n = 55)	Abnormal (n = 36)	
Gestational age at birth, median (IQR), wk	38.7 (34.4-41.0)	38.4 (32.7-42.0)	.06
Preterm, <37 wk gestational age	5 (9)	7 (19)	.15
Male sex	29 (53)	20 (56)	.79
Cesarean delivery	31/45 (69)	24/31 (77)	.41
Emergency cesarean delivery	5/26 (19)	6/16 (38)	.19
Birth weight, mean (SD), g	3175 (410)	3154 (584)	.84
Low birth weight, <2500 g	4 (7)	4 (11)	.71
Macrosomia, >4000 g	1 (2)	2 (6)	.56
Weight percentiles			
Appropriate for gestational age	45 (82)	23 (64)	.05
Small for gestational age	4 (7)	7 (19)	.11
Large for gestational age	6 (11)	6 (17)	.43
NICU admission	7/54 (13)	11/36 (31)	.04
Preterm, 37 wk gestational age, (n = 12)	3/5 (60)	4/7 (57)	>.99
Term, ≥37 wk gestational age, (n = 78)	4/49 (8)	7/29 (24)	.05
NICU length of stay, median (IQR), d	2.0 (2-10)	6.5 (1-30)	.62
Postnatal microcephaly	0	6 (17)	.003

Abbreviations: IQR, interquartile range; NICU, neonatal intensive care unit.

^a^ Neonatal outcomes for all liveborn infants (n = 91). When data were missing, denominators are noted.

^b^ *P* values were 2-sided and calculated by the *t* test, Fisher exact, χ^2^, or Kruskal-Wallis test as appropriate. *P* values less than .05 were considered significant.

A composite adverse neonatal outcome was determined for each fetus in the abnormal ultrasonographic results group. Of the 92 mother-neonate dyads, 45 neonates (49%) had an adverse neonatal outcome (eTable 2 in the Supplement). There were 2 perinatal deaths. One fetal death occurred at 36 weeks’ gestation. In this case, maternal infection occurred at 25 weeks, and the pregnant woman had a normal ultrasonographic finding at 30 weeks, followed by a second ultrasonographic scan at 33 weeks that showed abnormal MCA Doppler measurements. The other was a neonatal death occurring on day 1 of life in an infant with multiple CNS anomalies, hydrocephalus, arthrogryposis, and severe fetal growth restriction. There were 41 of 91 neonates (45%) with abnormal results on neonatal examination. The most common abnormal findings on neonatal examination were hypertonia (n = 12), seizures (n = 8), hypotonia (n = 6), microcephaly (n = 6), congenital contractures (n = 3), and dysmorphic features (n = 3). Of the 64 infants who underwent a funduscopic eye examination, 8 (13%) were found to have ophthalmologic findings consistent with congenital Zika syndrome. Three of the 27 neonates had hearing loss when given a hearing test. Postnatal neuroimaging was performed in 68 neonates, of whom 23 (34%) had abnormal results. In this cohort of 92 neonates whose mothers had prenatal ultrasonography, there was no association between gestational age at delivery, gestational age at time of Zika virus infection, trimester of maternal Zika virus infection, and adverse neonatal outcomes (eTable 3 in the Supplement).

Associations between abnormal results on prenatal ultrasonography and abnormal results on neonatal examination, abnormal findings on postnatal neuroimaging, and composite adverse neonatal outcome were investigated (Table 3 and eTable 4 in the Supplement). After adjusting for maternal age and number of ultrasonographic examinations performed, a major Zika virus–associated abnormal finding on ultrasonography, which occurred in 11 of the 92 pregnant women (12%), was associated with significantly increased probability of abnormal results on neonatal examination (aOR, 11.6; 95% CI, 1.8-72.8), abnormal results on postnatal neuroimaging (aOR, 6.7; 95% CI, 1.1-38.9), and composite adverse neonatal outcome (aOR, 27.2; 95% CI, 2.5-296.6). Abnormalities of the CNS and fetal growth restriction were both associated with significantly increased risk of composite adverse neonatal outcome (aOR, 2.2; 95% CI, 2.4-301.5] for CNS abnormalities and 14.6; 95% CI, 1.2-174.0 for fetal growth restriction). Abnormal MCA Doppler measurements were associated with abnormal results on neonatal examination (aOR, 12.8; 95% CI, 2.6-63.2), postnatal neuroimaging (aOR, 8.8; 95% CI, 1.7-45.9), and composite adverse neonatal outcome (aOR, 20.5; 95% CI, 3.2-132.6). Oligohydramnios was associated with an increased risk of abnormal result on neonatal examination (aOR, 13.5; 95% CI, 1.1-170.3).

**Table 3.** Adjusted Associations Between Prenatal Ultrasonographic Result and Neonatal Outcomes Among Women Diagnosed During Pregnancy With Zika Virus Infection

Ultrasonographic Result	Neonatal Examination			Postnatal Neuroimaging				Composite Neonatal Outcome			
	Normal Result, No. (%) (n = 51)	Abnormal Result, No. (%) (n = 41)^a^	aOR (95% CI)	Normal Result, No. (%) (n = 45)	Abnormal Result, No. (%) (n = 23)	aOR (95% CI)^b^	*P* Value	Normal Result, No. (%) (n = 47)	Abnormal Result, No. (%) (n = 45)^a^	aOR (95% CI)^b^	*P* Value
Any abnormal finding (n = 37)	17 (33.3)	20 (48.8)	2.15 (0.9-5.3)	16 (35.6)	12 (52.2)	1.5 (0.5-4.4)		15 (31.9)	22 (48.9)	2.4 (1.0-5.8)	
Zika virus–associated abnormal finding (n = 11)^c^	2 (3.9)	9 (22.0)	11.6 (1.8-72.8)	2 (4.4)	7 (30.4)	6.7 (1.1-38.9)		1 (2.1)	10 (22.2)	27.2 (2.5-296.6)	
CNS abnormality (n = 10)	2 (3.9)	8 (19.5)	11.0 (1.7-73.4)	1 (2.2)	7 (30.4)	13.9 (1.4-140.0)		1 (2.1)	9 (20.0)	27.2 (2.4-310.5)	
Microcephaly (n = 7)	1 (2.0)	6 (14.6)	23.4 (1.8-296.5)	0	5 (21.7)	NA	.003^d^	1 (2.1)	6 (13.3)	17.8 (1.4-218.9)	
Calcifications (n = 9)	2 (3.9)	7 (17.1)	9.2 (1.4-61.6)	1 (2.2)	6 (26.1)	11.5 (1.1-118.1)		1 (2.1)	8 (17.8)	22.8 (2.0-257.2)	
Ventriculomegaly (n = 6)	1 (2.0)	5 (12.2)	12.8 (1.1-150.0)	0	5 (21.7)	NA	.003^d^	0	6 (13.3)	NA	.01^d^
Fetal growth restriction (n = 7)	1 (2.0)	6 (14.3)	18.7 (1.5-229.5)	1 (2.2)	4 (17.4)	4.9 (0.4-57.5)		1 (2.1)	6 (13.3)	14.6 (1.2-175.0)	
Ultrasonographic findings of unknown significance (n = 37)	17 (33.3)	20 (48.8)	2.2 (0.9-5.3)	16 (35.6)	12 (52.2)	1.5 (0.5-4.4)		15 (31.9)	22 (48.9)	2.4 (1.0-5.8)	
Without Zika virus–associated finding (n = 26)	15 (29.4)	11 (26.8)	0.9 (0.4-2.3)	14 (31.1)	5 (21.7)	0.6 (0.2-2.0)		14 (29.8)	12 (26.7)	0.9 (0.4-2.2)	
Abnormal Doppler results (n = 17)	4 (7.8)	13 (31.7)	7.0 (2.0-31.3)	4 (8.9)	9 (39.1)	4.6 (1.1-20.5)		4 (8.5)	13 (28.9)	6.0 (1.6-23.5)	
Umbilical artery (n = 5)	2 (3.9)	3 (7.3)	2.2 (0.3-14.1)	2 (4.4)	2 (8.7)	1.3 (0.2-11.7)		2 (4.3)	3 (6.7)	1.8 (0.3-11.6)	
Middle cerebral artery (n = 16)	3 (5.9)	13 (31.7)	12.8 (2.6-63.2)	3 (6.7)	10 (43.5)	8.8 (1.7-45.9)		2 (4.3)	14 (31.1)	20.5 (3.2-132.6)	
Fluid abnormalities (n = 13)	5 (9.8)	8 (19.5)	2.4 (0.7-8.9)	6 (13.3)	6 (26.1)	1.4 (0.3-5.7)		5 (10.6)	8 (17.8)	2.0 (0.5-7.1)	
Oligohydramnios (n = 6)	1 (2.0)	5 (12.2)	13.5 (1.1-170.3)	2 (4.4)	4 (17.4)	2.2 (0.2-18.2)		1 (2.1)	5 (11.1)	10.8 (0.9-133.7)	
Polyhydramnios (n = 7)	4 (7.8)	3 (7.3)	0.9 (0.2-4.2)	4 (8.9)	2 (8.7)	1.0 (0.2-6.1)		4 (8.5)	3 (6.7)	0.8 (0.2-3.6)	

Abbreviations: aOR, adjusted odds ratio; CNS, central nervous system; NA, not available.

^a^ The stillbirth case was included and considered to have an abnormal finding on neonatal examination and composite adverse neonatal outcome.

^b^ The aORs and 95% CIs were calculated by logistic regression, adjusting for maternal age and number of ultrasonographic examinations.

^c^ Zika virus–associated CNS anomalies included microcephaly, calcifications, ventriculomegaly, Blake pouch cyst, cerebellar vermian hypoplasia, and agenesis of the corpus callosum. Most common findings are shown. See eTable 4 in the Supplement for complete results.

^d^ When ORs were unable to be calculated, the Fisher exact test was performed and 2-way *P* values are presented.

Table 4 shows the distribution of the mother-neonate dyads with abnormal results on prenatal ultrasonography (n = 37), abnormal results on neonatal examination (n = 41), and abnormal results on postnatal neuroimaging (n = 23). Of the 37 pregnant women with abnormal results on prenatal ultrasonography, 20 neonates (54%) had an abnormal result on neonatal examination and 27 neonates (73%) had at least 1 postnatal neuroimaging study, of whom 12 of the 27 neonates (44%) had an abnormal result. Two neonates had both normal results on prenatal ultrasonography and on neonatal examinations but abnormal results on postnatal neuroimaging: 1 neonate had a posterior fossa hemorrhage and the second neonate had cerebral calcifications that were not detected antenatally. Detailed descriptions of neonatal outcome for each case by mother-neonate dyad are shown in the supplemental material (eTable 5 in the Supplement).

**Table 4.** Distribution of Mother-Neonate Dyads by Abnormal Findings on Prenatal Ultrasonography, Neonatal Examination, and Postnatal Neuroimaging Among 92 Women Diagnosed During Pregnancy With Zika Virus Infection

Abnormal Findings	No. of Mother-Neonate Dyads
Prenatal ultrasonography	37
Ultrasonography only	15
Ultrasonography and neonatal examination	20
Ultrasonography and neonatal examination only	10
Ultrasonography and postnatal neuroimaging	12
Ultrasonography and postnatal neuroimaging only	2
Ultrasonography, neonatal examination, and postnatal neuroimaging	10
Neonatal examination	41
Neonatal examination only	12
Neonatal examination and postnatal neuroimaging	19
Neonatal examination and postnatal neuroimaging only	9
Postnatal neuroimaging	23
Postnatal neuroimaging only	2

In our cohort, an abnormal result on prenatal ultrasonography had a sensitivity of 48.9% (95% CI, 33.7%-64.2%) and a specificity of 68.1% (95% CI, 52.9%-80.1%) for association with a composite adverse neonatal outcome. For a major Zika-associated abnormal result on prenatal ultrasonography, the sensitivity was lower (22.2%; 95% CI, 11.2%-37.1%), but the specificity was higher (97.9%; 95% CI, 88.7%-99.9%). In this study of pregnant women with confirmed Zika virus infection, the positive predictive value of any abnormality on prenatal ultrasonography for composite adverse neonatal outcome was 59.5% (95% CI, 46.7%-71.0%) and the negative predictive value was 58.2% (95% CI, 49.6%-66.3%); for major, Zika-associated abnormal results, the positive and negative predictive values for composite adverse neonatal outcome were 90.9% (95% CI, 57.2%-98.7%) and 56.8% (95% CI, 52.8% to 60.7%), respectively.

## Discussion

In this study, we describe the spectrum of prenatal ultrasonographic findings in Zika virus–confirmed pregnancies and evaluate the associations with neonatal examination and postnatal neuroimaging abnormalities. We found that major Zika virus–associated abnormalities observed on prenatal ultrasonography were associated with a 6-fold to 27-fold increase in the odds of composite adverse neonatal outcomes of abnormal results on neonatal examination or postnatal neuroimaging. Specific ultrasonographic findings associated with adverse neonatal outcome include CNS abnormalities, cerebral calcifications, ventriculomegaly, fetal growth restriction, and abnormal MCA Doppler measurements. The sensitivity of prenatal ultrasonography to predict overall composite adverse neonatal outcomes was low (48.9%) because more than half of the patients with abnormal results on neonatal examinations had no structural findings on prenatal ultrasonography. This study adds to the current published literature that is, to date, primarily composed of retrospective studies of patients already with ultrasonographic findings suggestive of congenital Zika syndrome.

Our results suggest that prenatal ultrasonography may have a limited ability to provide reassurance of a normal neonatal outcome in maternal Zika virus infection. As we learn more about the spectrum of clinical sequelae of Zika virus infection, it has become clear that congenital Zika syndrome encompasses not only structural malformations (which may be identified by prenatal ultrasonography), but also disorders of neurologic function (which may not be evaluated on routine prenatal ultrasonography). Although previously of unknown significance in Zika virus infection, we have provided an analysis of ultrasonographic measurements that may have potential to be helpful in the identification of fetuses at risk for adverse neonatal outcomes. Although our numbers were small, abnormalities in MCA Doppler measurements and oligohydramnios were associated with adverse neonatal outcomes in our study sample. Monitoring of MCA Doppler measurements and amniotic fluid levels could be important components of fetal monitoring and surveillance. Future research in validating prenatal ultrasonography techniques that could provide clues to functional outcomes is clearly needed. The ability to predict functional outcomes prenatally could guide patient counseling and decision making regarding the pregnancy as well as aid in delivery planning to ensure optimal support services for a potentially affected neonate. As with cerebral palsy, neurodevelopmental outcomes may not manifest in the neonatal period,^27^ and data on neurobehavioral outcomes in congenital Zika syndrome remain unknown.

Recently, Pomar et al^13^ reported on the spectrum of ultrasonographic findings in an observational cohort of Zika virus–positive patients and found a 9.0% rate of CNS abnormalities and a 1.7% rate of microcephaly. Rates of major CNS abnormalities were similar to those observed in our study; however, other differences between both studies (including a higher rate of microcephaly in our cohort) may be due to varying definitions of Zika virus positivity. All the patients in our cohort were confirmed by serum or urine RT-PCR, whereas Pomar et al included serologic tests to diagnose Zika virus, which may be confounded by cross-reactivity with other arboviruses. Our study only evaluated women who were symptomatic during pregnancy, whereas the study by Pomar et al performed Zika virus screening in pregnant women, of whom only 17.3% (52 of 301 pregnant women) were symptomatic. More prospective data are needed to clarify the Zika virus spectrum of disease and transmission risk in symptomatic vs asymptomatic infections.

### Strengths and Limitations

Our study had several strengths. This is a well-characterized prospective cohort of pregnant women and their neonates. All prenatal and postnatal evaluations were conducted at a single institution; a single protocol was followed by the same group of investigators who examined every patient, with the same set of diagnostic studies performed on all patients in the same laboratory. This greatly reduced variability in practices and definitions used in the study, thus favoring reproducibility of results. Prenatal ultrasonographic studies were interpreted prior to delivery and neonatal outcomes obtained shortly after delivery. Second, maternal Zika virus infection was confirmed molecularly by RT-PCR at the time of acute infection. In addition, the presence of other congenital infections (which could lead to CNS findings) was rigorously excluded in all patients.

Our study also had several limitations. One potential limitation is that this cohort is derived from patients at a single study site in Brazil, which may limit the generalizability of our findings to other regions that have reported varying rates of birth defects from Zika virus infection.^1^ Because this was an observational cohort, many women enrolled in the parent study declined prenatal ultrasonographic examination, citing the burden of traveling to the obstetrical facility (although all were offered transportation from their homes) or fear of possible fetal abnormalities related to Zika virus infection.^2^ This could contribute to selection bias in our cohort, although patient autonomy in decision making surrounding prenatal screening tests is a real-world consideration. Although most of the neonates in our cohort underwent transfontanelle ultrasonography, this study is unable to compare the relative performance of computed tomography, magnetic resonance imaging, or transfontanelle ultrasonography in identifying clinically significant lesions because not every infant underwent each modality. However, we did not find that the prenatal identification of an ultrasonographic abnormality was associated with increased use of postnatal neuroimaging in our study population. Neonates with concerning findings on neonatal examination were more likely to undergo further evaluation with postnatal neuroimaging.

## Conclusions

In the setting of confirmed Zika virus infection in pregnancy, prenatal ultrasonography is a useful tool for anticipating an association with adverse neonatal outcomes, but its negative predictive value for adverse neonatal outcomes is low. This information is critical for patient counseling and to prepare clinicians to optimize postnatal care and follow-up for Zika virus–exposed pregnancies.

## Notes

Supplement. **eTable 1.** Patient Demographics **eTable 2.** Classification of Adverse Neonatal Outcomes **eTable 3.** Association Between Trimester of Infection and Neonatal Outcome **eTable 4.** Unadjusted Associations Between Prenatal Ultrasound and Neonatal Outcomes **eTable 5.** Detailed Neonatal Outcomes by Mother-Neonate Dyad
