# Supplementary material for: Association of Prenatal Ultrasonographic Findings With Adverse Neonatal Outcomes Among Pregnant Women With Zika Virus Infection in Brazil
Source: JAMA Netw Open. 2018 Dec 28;1(8):e186529. doi: 10.1001/jamanetworkopen.2018.6529 (PMC6324324; doi:10.1001/jamanetworkopen.2018.6529)
Supplement: Supplement. — eTable 1. Patient Demographics eTable 2. Classification of Adverse Neonatal Outcomes eTable 3. Association Between Trimester of Infection and Neonatal Outcome eTable 4. Unadjusted Associations Between Prenatal Ultrasound and Neonatal Outcomes eTable 5. Detailed Neonatal Outcomes by Mother-Neonate Dyad [file jamanetwopen-1-e186529-s001.pdf]

Supplementary Online Content

Pereira JP Jr, Nielsen-Saines K, Sperling J, et al. Association of prenatal ultrasonographic findings with adverse neonatal outcomes among pregnant women with Zika virus infection in Brazil. *JAMA Netw Open*. 2018;1(8):e186529. doi:10.1001/jamanetworkopen.2018.6529

eTable 1. Patient Demographics
eTable 2. Classification of Adverse Neonatal Outcomes
eTable 3. Association Between Trimester of Infection and Neonatal Outcome
eTable 4. Unadjusted Associations Between Prenatal Ultrasound and Neonatal Outcomes
eTable 5. Detailed Neonatal Outcomes by Mother-Neonate Dyad

This supplementary material has been provided by the authors to give readers additional information about their work.

© Pereira JP et al. *JAMA Network Open*.

eTable 1. Patient Demographics

	Prenatal ultrasound		*p*
	Normal (n=55)	Abnormal (n=37)	
Maternal age (years)	30.5 ± 6.1	27.8 ± 6.5	0.05
Advanced maternal age (≥ 35 years)	13 (23.6%)	5 (13.5%)	0.23
No. of ultrasound examinations performed	2.6 ± 1.3	3.5 ± 1.9	0.01
Gestational age at infection (median weeks (range))	18.3 (4.0-31.3)	14.6 (4.3-35.1)	0.11
First trimester infection (< 14 weeks, n=30)	14 (25.5%)	16 (43.2%)	
Second trimester infection (14 to 27 weeks, n=59)	40 (72.7%)	19 (51.3%)	0.07
Third trimester infection (≥ 28 weeks, n=3)	1 (1.8%)	2 (5.4%)	

Data presented as mean ± standard deviation or n (%) unless otherwise noted. P values are two-sided and calculated by t-test, chi-squared, Fisher's exact test, or Kruskal-Wallis as appropriate. P values < 0.05 are considered significant.

© Pereira JP et al. *JAMA Network Open*.

eTable 2. Classification of Adverse Neonatal Outcomes

	n	%
**Abnormal outcomes**	45/92	48.9
**Perinatal death**	2	2.2
Fetal demise	1	1.1
Neonatal demise (<28 days of life)	1	1.1
**Abnormal neonatal examination***	41/91	45.1
Physical examination	39	42.4
Fundoscopic exam of eye	8/64	12.5
Hearing exam	3/27	11.1
**Abnormal postnatal Imaging**	23/68	33.8
Transfontanelle ultrasound	11/62	17.7
Head CT	7/17	41.2
Head MRI	17/40	42.5

Data is presented as n (%). Not all infants underwent each examination or study; the denominator for each is indicated. CT, computed tomography; MRI, magnetic resonance imaging. Abnormal neonatal outcomes include perinatal death, abnormal neonatal examination, and/or abnormal imaging findings.

© Pereira JP et al. *JAMA Network Open*.

eTable 3. Association Between Trimester of Infection and Neonatal Outcome

	Neonatal examination			Postnatal Neuroimaging			Composite Neonatal Outcome		
	Normal (n=51)	Abnormal (n=41)	*p*	Normal (n=45)	Abnormal (n=23)	*p*	Normal (n=47)	Abnormal (n=45)	*p*
Gest. age at birth	39.0 (38.0 to 39.1)	38.2 (37.9 to 39.3)	0.29	38.7 (38.0 to 39.6)	38.6 (37.1 to 39.3)	0.40	39.0 (38.0 to 39.1)	38.2 (37.5 to 39.3)	0.27
Gest. age at infection	16.0 (10.9 to 20.6)	18.1 (13.6 to 22.4)	0.15	18.3 (15.3 to 23.3)	14.6 (9.3 to 21.4)	0.13	15.7 (10.9 to 20.6)	18.0 (13.6 to 22.4)	0.13
1st trimester (n=30)	19 (37.3%)	11 (26.8%)	0.45	10 (22.2%)	9 (39.1%)	0.20	18 (38.3%)	12 (26.7%)	0.48
2nd trimester (n=59)	31 (60.8%)	28 (69.3%)		32 (71.1%)	14 (60.9%)		28 (59.6%)	31 (68.9%)	
3rd trimester (n=3)	1 (2.0%)	2 (4.9%)		3 (6.7%)	0 (0%)		1 (2.1%)	2 (4.4%)	

Data are presented as median weeks (IQR) or n (%). P values are two-sided and calculated from Kruskal-Wallis or Fisher exact tests as appropriate. P values < 0.05 were considered significant. Not all infants underwent each examination or study; the denominator for each is indicated. Abnormal composite neonatal outcomes include perinatal death, abnormal neonatal examination, and/or abnormal imaging findings. The stillbirth case was included and considered to have an abnormal neonatal exam and abnormal composite neonatal outcome.

© Pereira JP et al. *JAMA Network Open*.

eTable 4. Unadjusted Associations Between Prenatal Ultrasound and Neonatal Outcomes

	Neonatal examination			Postnatal Neuroimaging			Composite Neonatal Outcome		
	Normal (n=51)	Abnormal (n=41)	OR (95% CI)	Normal (n=45)	Abnormal (n=23)	OR (95% CI)	Normal (n=47)	Abnormal (n=45)	OR (95% CI)
**Abnormal prenatal US (n=37)**	17 (33.3%)	20 (48.8%)	1.9 (0.8 to 4.4)	16 (35.6%)	12 (52.2%)	2.0 (0.7 to 5.5)	15 (31.9%)	22 (48.9%)	2.0 (0.9 to 4.8)
**Zika-associated US finding (n=11)**	2 (3.9%)	9 (22.0%)	6.9 (1.4 to 34.0)	2 (4.4%)	7 (30.4%)	9.4 (1.8 to 50.1)	1 (2.1%)	10 (22.2%)	13.1 (1.6 to 107)
CNS abnormality (n=10)	2 (3.9%)	8 (19.5%)	5.9 (1.2 to 29.8)	1 (2.2%)	7 (30.4%)	19.3 (2.2 to 168)	1 (2.1%)	9 (20.0%)	11.5 (1.4 to 95.0)
Microcephaly (n=7)	1 (2.0%)	6 (14.6%)	8.6 (1.0 to 74.4)	0	5 (21.7%)	0.003*	1 (2.1%)	6 (13.3%)	7.1 (0.8 to 61.3)
Calcifications (n=9)	2 (3.9%)	7 (17.1%)	5.0 (0.9 to 25.8)	1 (2.2%)	6 (26.1%)	15.5 (1.7 to 139)	1 (2.1%)	8 (17.8%)	10.0 (1.2 to 83.2)
Ventriculomegaly (n=6)	1 (2.0%)	5 (12.2%)	6.9 (0.8 to 62.0)	0	5 (21.7%)	0.003*	0	6 (13.3%)	0.01*
Blake pouch cyst (n=3)	0	3 (7.3%)	0.09*	0	3 (13.0%)	0.04*	0	3 (6.7%)	0.11*
Cerebellar vermis hypoplasia (n=3)	0	3 (7.3%)	0.09*	0	2 (8.7%)	0.11*	0	3 (6.7%)	0.13*
Agenesis of corpus callosum (n=2)	0	2 (4.9%)	0.20*	0	1 (4.4%)	0.14*	0	2 (4.4%)	0.24*
Fetal growth restriction (n=7)	1 (2.0%)	6 (14.3%)	8.6 (0.9 to 74.4)	1 (2.2%)	4 (17.4%)	9.3 (1.0 to 88.5)	1 (2.1%)	6 (13.3%)	7.1 (0.8 to 61.3)
Arthrogryposis (n=1)	0	1 (2.4%)	0.45*	0	1 (4.4%)	0.34*	0	1 (2.2%)	0.49*
**US findings of unknown significance (n=37)**	17 (33.3%)	20 (48.8%)	1.9 (0.8 to 4.4)	16 (35.6%)	12 (52.2%)	2.0 (0.7 to 5.5)	15 (31.9%)	22 (48.9%)	2.0 (0.9 to 4.8)
Without Zika-associated US finding (n=26)	15 (29.4%)	11 (26.8%)	0.9 (0.4 to 2.2)	14 (31.1%)	5 (21.7%)	0.6 (0.2 to 2.0)	14 (29.8%)	12 (26.7%)	0.9 (0.4 to 2.1)
Abnormal Doppler (n=17)	4 (7.8%)	13 (31.7%)	5.5 (1.6 to 18.4)	4 (8.9%)	9 (39.1%)	6.6 (1.8 to 24.8)	4 (8.5%)	13 (28.9%)	4.4 (1.3 to 14.7)
Umbilical artery (n=5)	2 (3.9%)	3 (7.3%)	1.9 (0.3 to 12.2)	2 (4.4%)	2 (8.7%)	2.1 (0.3 to 15.6)	2 (4.3%)	3 (6.7%)	1.6 (0.3 to 10.1)
Middle cerebral artery (n=16)	3 (5.9%)	13 (31.7%)	7.4 (2.0 to 28.3)	3 (6.7%)	10 (43.5%)	10.8 (2.6 to 45.1)	2 (4.3%)	14 (31.1%)	10.2 (2.2 to 47.9)
Fluid abnormalities (n=13)	5 (9.8%)	8 (19.5%)	2.2 (0.7 to 7.4)	6 (13.3%)	6 (26.1%)	2.3 (0.7 to 8.1)	5 (10.6%)	8 (17.8%)	1.8 (0.6 to 6.0)
Oligohydramnios (n=6)	1 (2.0%)	5 (12.2%)	6.9 (0.8 to 62.0)	2 (4.4%)	4 (17.4%)	4.5 (0.8 to 26.9)	1 (2.1%)	5 (11.1%)	5.8 (0.6 to 51.3)
Polyhydramnios (n=7)	4 (7.8%)	3 (7.3%)	0.9 (0.2 to 4.4)	4 (8.9%)	2 (8.7%)	1.0 (0.2 to 5.8)	4 (8.5%)	3 (6.7%)	0.8 (0.2 to 3.6)
Placentomegaly (n=11)	7 (13.7%)	4 (9.8%)	0.7 (0.2 to 2.5)	4 (8.9%)	3 (13.0%)	1.5 (0.3 to 7.5)	7 (14.9%)	4 (8.9%)	0.6 (0.2 to 2.1)
Macrosomia (n=8)	5 (9.8%)	3 (7.3%)	0.7 (0.2 to 3.2)	3 (6.7%)	2 (8.7%)	1.3 (0.2 to 8.6)	5 (10.6%)	3 (6.7%)	0.6 (0.1 to 2.7)
Mega cisterna magna (n=4)	1 (2.0%)	3 (7.3%)	4.0 (0.4 to 39.5)	1 (2.2%)	2 (8.7%)	4.2 (0.4 to 48.9)	1 (2.1%)	3 (6.7%)	3.3 (0.3 to 32.8)

Data are presented as n (%). Unadjusted odds ratios and 95% confidence intervals were calculated by logistic regression. When odds ratios were unable to be calculated, Fisher's exact test was performed and two-way p values are presented and indicated with asterisk (*). Values were considered statistically significant if $p<0.05$. The stillbirth case was included and considered to have an abnormal neonatal exam and abnormal composite neonatal outcome.
Abbreviations: US, ultrasound; OR, odds ratio; CI, confidence interval; CNS, central nervous system

© Pereira JP et al. *JAMA Network Open.*

eTable 5. Detailed Neonatal Outcomes by Mother-Neonate Dyad

Dyad No.	Gest. Age Infection (weeks)	Gest. Age 1st US (Weeks)	Time from Infection to 1st US (Weeks)	No. of Prenatal US	Abnormal Finding on US	Gest. Age Birth	Abnormal Neonatal Exam	Abnormal Neonatal Imaging	Imaging Modality	NICU Admission	Adverse Neonatal Outcome Composite	Outcome
1*	18.29	21.71	3.42	3	Yes	36.86	No	No	US, MRI	Yes	No	NICU admission 30 days
2*	30.71	34.00	3.29	1	Yes	38.71	No	No	US	No	No	
3*	31.29	33.86	2.57	1	No	37.86	Yes	No	US	No	Yes	Hypertonia, polydactyly
4*	14.00	17.86	3.86	1	No	39.43	Yes	Yes	US, MRI	No	Yes	Cortical thumb, hyperreflexia
5*	13.57	19.00	5.43	4	No	39.14	Yes	No	US	No	Yes	Abnormal neurologic exam, hypertonia
6*	21.14	28.00	6.86	2	No	38.00	Yes	Yes	US, CT, MRI	No	Yes	Hyperreflexia, hypertonia, clonus
7*	9.57	15.57	6.00	4	No	39.29	Yes	Yes	CT, MRI	Yes	Yes	Abnormal neurologic exam, seizures
8*	14.43	19.71	5.28	4	Yes	38.57	Yes	Yes	US, MRI	No	Yes	Cortical thumb, hypertonia
9*	14.00	19.14	5.14	3	Yes	35.57	Yes	No	US	No	Yes	Abnormal neurologic exam
10*	25.57	30.71	5.14	2	Yes		Yes				Yes	Stillbirth at 36 weeks
11*	16.00	22.00	6.00	1	No	37.86	No	No	US, MRI	No	No	
12*	22.29	28.71	6.42	5	Yes	32.71	Yes	Yes	US, MRI	Yes	Yes	Microcephaly, small for gestational age, eye lesions, abnormal neurologic exam
13*	10.86	17.00	6.14	4	No	38.86	No	No	MRI	No	No	
14*	20.43	27.86	7.43	3	No	40.71	No	No	US	No	No	
15*	23.00	28.71	5.71	1	No	39.14	No	No	US	No	No	Hyperreflexia, excess scalp
16*	25.57	30.71	5.14	2	No	38.71	Yes	No	US	No	Yes	Brachycephaly, dysmorphic features
17*	22.00	26.57	4.57	3	Yes	39.86	No	No	US, MRI	No	No	
18*	22.43	27.86	5.43	3	No	38.00	Yes	No	US, CT, MRI	No	Yes	Eye abnormalities, hyperreflexia, abnormal neurologic exam
19*	8.00	35.71	27.71	3	Yes	39.29	Yes	Yes	US, CT, MRI	Yes	Yes	Microcephaly, hypertonia, seizures
20*	18.86	25.00	6.14	3	No	39.00	No	No	US, MRI	No	No	
21*	16.29	21.43	5.14	3	Yes	37.00	Yes		None	No	Yes	Seizures, abnormal neurologic exam
22*	19.71	25.86	6.15	3	No	38.00	Yes	No	US	No	Yes	Eye abnormalities
23*	35.14	40.43	5.29	1	Yes	40.57	Yes	No	US, CT	Yes	Yes	Hypotonia, abnormal neurologic exam

© Pereira JP et al. *JAMA Network Open*.

Dyad No.	Gest. Age Infection (weeks)	Gest. Age 1st US (Weeks)	Time from Infection to 1st US (Weeks)	No. of Prenatal US	Abnormal Finding on US	Gest. Age Birth	Abnormal Neonatal Exam	Abnormal Neonatal Imaging	Imaging Modality	NICU Admission	Adverse Neonatal Outcome Composite	Outcome
24*	12.00	29.14	17.14	8	Yes	42.00	Yes	Yes	US, CT, MRI	Yes	Yes	Microcephaly, hypertonia, arthrogryposis
25	8.14	12.29	4.15	5	Yes	38.71	Yes	Yes	US, CT, MRI	No	Yes	Abnormal neurologic exam
26*	16.57	22.71	6.14	3	No	38.00	Yes	No	US, MRI	No	Yes	Cortical thumb, abnormal neurologic exam
27*	16.43	24.86	8.43	3	No	39.29	Yes	No	US	No	Yes	Abnormal neurologic exam, clenched fists
28*	23.43	30.71	7.28	3	No	39.14	No	No	US	No	No	
29	4.00	14.71	10.71	5	No	40.29	No	No	US	No	No	
30*	26.00	33.43	7.43	1	No	40.57	Yes	No	US, CT	No	Yes	Abnormal neurologic exam, hyperexcitable
31*	14.57	21.71	7.14	4	Yes	35.57	Yes	Yes	US, CT, MRI	Yes	Yes	Hypertrophic cardiomyopathy, hypoactive
32*	18.00	24.43	6.43	2	No	37.14	No	Yes	US, CT, MRI	Yes	Yes	
33	22.57	28.29	5.72	3	No	39.86	No	No	US	No	No	
34*	23.29	32.00	8.71	1	Yes	40.14	Yes	No	US	Yes	Yes	Small for gestational age, abnormal neurologic exam, posturing, hearing abnormalities
35*	25.43	35.43	10.00	1	No	39.57	No	No	US	No	No	
36*	26.71	35.86	9.15	1	Yes	35.57	Yes	No	US	Yes	Yes	Small for gestational age
37	12.29	19.29	7.00	2	Yes	36.00	No	No	MRI	No	No	
38*	26.71	35.29	8.58	2	Yes	39.14	No	Yes	US, CT	No	Yes	
39*	21.00	30.29	9.29	3	Yes	39.57	Yes	No	US	No	Yes	Abnormal neurologic exam, hypertonia
40*	8.57	17.86	9.29	6	Yes	38.29	Yes	Yes	US, CT	No	Yes	Congenital hip dislocation
41*	17.43	30.29	12.86	2	Yes	35.71	Yes	No	US	No	Yes	Sacral dimple, abnormal neurologic exam
42	7.71	21.57	13.86	5	No	39.00	No		None	No	No	
43	17.29	23.29	6.00	5	No	38.57	No	No	US, MRI	No	No	
44	24.00	31.71	7.71	2	No	39.14	No		None	No	No	
45*	18.14	28.86	10.72	2	Yes	38.57	Yes	Yes	US, MRI	No	Yes	Abnormal neurologic exam, hypotonia, hyperreflexia, hyperexcitability
46*	15.29	24.71	9.42	4	No	38.29	No	No	US	Yes	No	Meconium aspiration syndrome
47*	13.29	21.86	8.57	5	Yes	39.00	No		None	No	No	

© Pereira JP et al. *JAMA Network Open*.

Dyad No.	Gest. Age Infection (weeks)	Gest. Age 1st US (Weeks)	Time from Infection to 1st US (Weeks)	No. of Prenatal US	Abnormal Finding on US	Gest. Age Birth	Abnormal Neonatal Exam	Abnormal Neonatal Imaging	Imaging Modality	NICU Admission	Adverse Neonatal Outcome Composite	Outcome
48*	26.71	35.71	9.00	1	No	38.00	Yes	Yes	US, MRI	No	Yes	Abnormal neurologic exam
49*	11.57	21.43	9.86	4	Yes	38.00	No	No	US, MRI	No	No	
50*	24.29	34.00	9.71	1	No	38.00	No	No	US, MRI	No	No	
51	7.29	16.71	9.42	4	Yes	38.29	No	No	US, MRI	Yes	No	Abnormal neurologic exam
52*	18.71	28.43	9.72	2	No	38.00	Yes	No	US, MRI	No	Yes	Abnormal neurologic exam
53	21.00	29.14	8.14	3	No	37.43	No		None	No	No	
54*	27.29	36.43	9.14	1	No	40.14	No	No	US	No	No	
55	5.57	15.43	9.86	5	No	40.57	No		None	No	No	Small for gestational age
56*	22.14	30.57	8.43	2	No	38.57	Yes	Yes	US, MRI	No	Yes	Abnormal neurologic exam, hypertonia
57	5.29	15.00	9.71	4	Yes	39.00	No	No	US, MRI	No	No	
58	17.43	24.57	7.14	3	Yes	41.57	No	Yes	US, CT, MRI	No	Yes	Small for gestational age, abnormal neurologic exam
59	7.57	17.14	9.57	3	No	40.43	No		None	No	No	
60*	17.00	34.71	17.71	2	Yes	38.00	Yes	No	MRI	No	Yes	Arthrogryposis, abnormal neurologic exam
61*	12.86	21.57	8.71	3	No	34.43	Yes	Yes	US, MRI	Yes	Yes	
62*	20.86	28.00	7.14	3	No	39.00	No	No	US	No	No	
63*	18.29	25.43	7.14	1	No	38.57	Yes		None	No	Yes	Laryngomalacia
64	5.29	15.00	9.71	3	No	34.43	Yes		None	Yes	Yes	Abnormal neurologic exam, hypotonia
65*	18.14	21.43	3.29	3	No	36.00	No	No	US	Yes	No	
66	5.43	15.57	10.14	5	No	39.00	No	No	US	No	No	
67*	21.43	30.43	9.00	4	No	40.14	Yes	Yes	US, CT, MRI	Yes	Yes	Seizures, abnormal neurologic exam
68*	10.86	22.14	11.28	5	Yes	37.00	No	No	MRI	No	No	
69*	27.00	34.71	7.71	2	No	41.00	Yes	No	US	No	Yes	
70	4.29	11.86	7.57	3	Yes	39.71	No	No	MRI	Yes	No	
71	15.71	27.71	12.00	3	Yes	40.71	No		None	No	No	
72	18.29	31.86	13.57	1	No	38.43	No	No	US	No	No	
73	8.86	21.14	12.28	4	No	38.00	No		None	No	No	
74	12.14	25.00	12.86	4	No	38.71	No		None	No	No	

© Pereira JP et al. *JAMA Network Open*.

Dyad No.	Gest. Age Infection (weeks)	Gest. Age 1st US (Weeks)	Time from Infection to 1st US (Weeks)	No. of Prenatal US	Abnormal Finding on US	Gest. Age Birth	Abnormal Neonatal Exam	Abnormal Neonatal Imaging	Imaging Modality	NICU Admission	Adverse Neonatal Outcome Composite	Outcome
75	14.29	22.00	7.71	4	No	37.00	No		None	No	No	
76	20.00	35.71	15.71	1	Yes	39.14	No		None	No	No	
77*	23.86	36.43	12.57	1	No	37.86	Yes	Yes	US, MRI	No	Yes	Abnormal neurologic exam
78	9.29	22.00	12.71	10	Yes	38.00	Yes	Yes	US, CT, MRI	No	Yes	Microcephaly, abnormal neurologic exam
79*	19.00	36.00	17.00	1	No	37.00	Yes	Yes	MRI	No	Yes	Abnormal neurologic exam, hypertonia of limbs, axial hypotonia, hyperreflexia
80	6.00	15.71	9.71	4	No	35.29	No	Yes	US, CT	No	Yes	
81*	17.29	30.43	13.14	1	No	38.00	No	No	US	No	No	
82	11.43	23.29	11.86	4	Yes	38.00	No		None	No	No	
83	9.14	26.29	17.15	6	Yes	40.00	Yes	Yes	US, CT, MRI	No	Yes	Microcephaly, abnormal neurologic exam, seizures
84	15.29	26.71	11.42	4	No	39.00	No		None	No	No	
85	15.29	22.29	7.00	4	Yes	37.00	No		None	No	No	
86*	19.00	37.43	18.43	1	No	35.00	No		None	No	No	
87	10.86	23.43	12.57	3	Yes	38.00	No		None	No	No	
88	10.14	33.14	23.00	1	No	39.00	No		None	No	No	
89	20.57	33.71	13.14	1	No	37.86	No		None	No	No	
90	14.57	27.43	12.86	2	No	39.00	No		None		No	
91	5.57	29.86	24.29	3	Yes	38.00	Yes		None	Yes	Yes	Arthrogryposis, microcephaly, multiple brain abnormalities, hydrocephalus, neonatal death on day of life 1.
92	16.86	35.14	18.28	2	No	40.43	No	No	US	No	No	

Gestational age (Gest. Age) is presented as weeks. Asterisks (*) indicated cases that were previously reported in Brasil, et al, NEJM, 2016 (patient numbers have changed).

Abbreviations: No., number; US, ultrasound; CT, computed tomography; MRI, magnetic resonance imaging; NICU, neonatal intensive care unit; OR, odds ratio; CI, confidence interval; CNS, central nervous system

© Pereira JP et al. *JAMA Network Open*.
